# Genetic Screening Revealed Latent Keratoconus in Asymptomatic Individuals

**DOI:** 10.3389/fcell.2021.650344

**Published:** 2021-05-31

**Authors:** Shihao Chen, Xing-Yong Li, Jia-Jia Jin, Ren-Juan Shen, Jian-Yang Mao, Fei-Fei Cheng, Zhen-Ji Chen, Emmanouela Linardaki, Stavroula Voulgaraki, Ioannis M. Aslanides, Zi-Bing Jin

**Affiliations:** ^1^Center for Refractive Surgery, The Eye Hospital, Wenzhou Medical University, Wenzhou, China; ^2^Division of Ophthalmic Genetics, The Eye Hospital, Wenzhou Medical University, National Center for International Research in Regenerative Medicine and Neurogenetics, Wenzhou, China; ^3^Beijing Institute of Ophthalmology, Beijing Tongren Eye Center, Beijing Tongren Hospital, Capital Medical University, Beijing Ophthalmology and Visual Sciences Key Laboratory, Beijing, China; ^4^DNA Analysis, Molecular Biology and Genetics Center, Heraklion, Greece; ^5^Emmetropia Mediterranean Eye Institute, Heraklion, Greece; ^6^Beijing Advanced Innovation Center for Big Data-Based Precision Medicine, Beihang University and Capital Medical University, Beijing Tongren Hospital, Beijing, China

**Keywords:** keratoconus, genetics, latent, asymptomatic, refractive surgery

## Abstract

**Purpose:**

To adopt molecular screening in asymptomatic individuals at high risk of developing keratoconus as a combinative approach to prevent subclinical patients from post-refractive surgery progressive corneal ectasia.

**Methods:**

In this study, 79 Chinese and nine Greek families with keratoconus were recruited, including 91 patients with clinically diagnosed keratoconus as well as their asymptomatic but assumptive high-risk first-degree relatives based on underlying genetic factor. Mutational screening of *VSX1*, *TGFBI*, and *ZEB1* genes and full clinical assessment including Pentacam Scheimpflug tomography were carried out in these individuals.

**Results:**

Five variants in *VSX1* and *TGFBI* genes were identified in three Chinese families and one Greek family, and four of them were novel ones. Surprisingly, ultra-early corneal changes in Belin/Ambrosio Enhanced Ectasia Display of Pentacam corneal topography together with co-segregated variants were revealed in the relatives who had no self-reported symptoms.

**Conclusions:**

Variants of *VSX1* and *TGFBI* genes identified in both the clinically diagnosed and subclinical patients may cause the keratoconus through an autosomal dominant inheritance pattern, with different variable expressivity. Combining genetic with Belin/AmbrosioEnhanced Ectasia Display can be used to identify patients with latent keratoconus. This study indicates that genetic testing may play an important supplementary role in re-classifying the disease manifestation and evaluating the preoperative examination of refractive surgery.

## Introduction

Keratoconus (KC, OMIM 148300) is a bilateral non-inflammatory ectasia disorder, sometimes asymmetric and characterized by progressive thinning, bulging, and a conical protrusion of the cornea (Krachmer et al., 1984; Rabinowitz, 1998). It is a relatively common disease with a prevalence ranging from 0.3 to 2,300 per 100,000, affecting both genders and diverse populations around the world (Gokhale, 2013), with higher frequency among certain ethnicities such as in Asians and Middle Easterns (Kok et al., 2012; Gokhale, 2013). It has been known that both genetic and environmental factors contribute to the disease occurrence (Gomes et al., 2015). Although most KC cases are sporadic, the incidence of familial aggregation in KC patients ranges from 6 to 23.5%, and first-degree relatives of KC patients have higher prevalence than the general population. High concordance of KC has been reported in studies of monozygotic twins. Most studies suggest an autosomal dominant mode of inheritance with different variable expressivity (Wang et al., 2000; Rabinowitz, 2003; Romero-Jimenez et al., 2010). These findings support the dominant role of a genetic factor in the pathogenesis of KC.

Over the years, many studies have postulated multiple chromosomal regions and genes (e.g., 5q31, 15q22.32-24.2, *VSX1*, *TGFBI*, *ZEB1*, *MIR184*, *SOD1*, and *ZNF469*) to be causative; therefore, KC is suspected to be a genetically heterogeneous disease (Rabinowitz, 2003; Valgaeren et al., 2018). The *VSX1* (OMIM 605020) gene is the most extensively studied gene as a candidate KC gene. An association has been established between *VSX1* gene and the pathogenesis of posterior polymorphous dystrophy (Bykhovskaya et al., 2016). Although the role of *VSX1* gene in KC remains controversial, various *VSX1* gene variants have been identified to be associated with KC in different populations (Shetty et al., 2015; Bykhovskaya et al., 2016; Karolak et al., 2016). It is reported that the expression of *VSX1* has been detected in adult corneas during corneal wound healing (Barbaro et al., 2006). The second candidate KC gene is the *TGFBI* (OMIM 601692) gene, which is responsible for Fuchs endothelial corneal dystrophy. TGFβ1 is reported to be involved in corneal scar formation and fibrosis (Zahir-Jouzdani et al., 2018), and recently, a study found that downregulation of the core elements of the TGF-β pathway influences corneal organization in KC cornea (Kabza et al., 2017). Recently, several mutations in *TGFBI* were identified in Chinese and Polish KC patients, indicating a potential role of *TGFBI* gene in KC pathogenesis (Guan et al., 2012; Bykhovskaya et al., 2016; Karolak et al., 2016). The *ZEB1* (OMIM 189909) gene is also a candidate gene of KC. This gene has been repeatedly reported in corneal dystrophy. Lechner et al. and Mazzotta et al. have identified the same missense variant c.1920G > T of *ZEB1* gene in different patients with KC and other corneal dystrophies, suggesting a potentially significant role of *ZEB1* gene in KC pathogenesis. The above three genes (*VSX1*, *TGFBI*, and *ZEB1*) have been widely studied and have been reported to be related to KC as well as other corneal genetic diseases. Consequently, we selected the above three genes as the target genes for preliminary molecular sequencing, for exploring genetic etiology of KC patients in this study.

In the present study, both genetic and clinical examinations were carried out in clinically diagnosed patients and all relatives. We focused on the genetic etiology and clinical phenotype of asymptomatic relatives, who were at high risk of developing KC, to explore whether molecular screening could be adopted as a combined approach to prevent subclinical patients from post-refractive surgery progressive corneal ectasia.

## Materials and Methods

This multicenter descriptive cross-sectional study was approved by the Institutional Review Board of The Eye Hospital of Wenzhou Medical University and The Emmetropia Mediterranean Eye Institute. The study adhered to the tenets of the Declaration of Helsinki. Written informed consent was obtained from all subjects prior to this study.

### Participant Recruitment

For this study, participants were recruited through patients who were admitted to The Eye Hospital of Wenzhou Medical University or The Emmetropia Mediterranean Eye Institute. A total of 88 families with KC, including 79 Chinese and nine Greek families, were recruited. Each family included a proband with clinically diagnosed KC and first-degree relatives of the proband; the parents of the proband must be included at least, regardless of whether they were asymptomatic or diagnosed as having KC. All the subjects were diagnosed by specialists from China and Greece, respectively. The diagnosis of KC is based on the following criteria: (1) at least one typical clinical feature of KC (e.g., regional stromal thinning, Vogt striae, Fleischer ring, Munson sign); (2) inferior–superior (I-S) index > 1.5, maximum keratometry (Kmax) > 47 D, and the difference in Kmax between the two eyes > 1 D; and (3) abnormal topographic criteria such as asymmetric bow tie, central or inferior steepening (Rabinowitz and McDonnell, 1989).

### Mutational Screening

Genomic DNA was isolated from peripheral blood and bidirectional sequencing of all coding regions of *VSX1* (including two additional novel exons) (Hosseini et al., 2008), *TGFBI*, and *ZEB1* genes was screened as previously described (Rao et al., 2017). The primers were designed based on the sequence information from the University of California, Santa Cruz (UCSC) genome browser (Supplementary Table 1). Genetic co-segregation of identified variants was confirmed in the families.

### Computational Assessment

To assess the functional effect of the identified variants, we employed a series of computational tools as described previously (Huang et al., 2015, 2017; Jin et al., 2017, 2018; Rao et al., 2017), including MutationTaster^1^ (Schwarz et al., 2014), Polyphen–2^2^ (Adzhubei et al., 2010), and SIFT^3^ (Kumar et al., 2009). In addition, minor allele frequency (MAF) of variants was obtained from 1,000 Genome (1000G) database, Exome Aggregation Consortium (ExAC) database, and Genome Aggregation Database (gnomAD) (Genomes Project et al., 2010; Lek et al., 2016). To evaluate the conservation of variants, multiple-sequence alignment of *TGFBI* in different species was produced by Clustal X (Version 2.1) and edited by BioEdit (Version 7.2.5.0), and amino acid sequences were obtained from National Center for Biotechnology Information^4^. Furthermore, protein structures of mutant and wild-type proteins of *TGFBI* were modeled by Phyre2^5^ and visualized by PyMOL (version 2.7) (Kelley et al., 2015).

### Whole-Exome Sequencing

Whole-exome sequencing (WES), as a complementary validation method, was performed on the samples of patients where variants in *VSX1*, *TGFBI*, and *ZEB1* genes were identified by Sanger sequencing. For each individual, at least 3 μg genomic DNA was broken into fragments to generate the Illumina libraries. The whole-exome region libraries were enriched by using an Exome Enrichment V5 Kit (Agilent Technologies, United States) following the manufacturer’s protocol and were sequenced by using paired-end 100–300 bp reads on a HiSeq 2000 sequencer (Illumina, United States). After quality control, WES sequence data were mapped to the reference human genome (hg19). Single nucleotide variants and indel variants were identified by GATK and annotated by ANNOVAR. For variant analyses, we summarized 40 candidate genes related to KC (Supplementary Table 2), based on several genetics reviews of KC and PubMed database (Bykhovskaya et al., 2016; Loukovitis et al., 2018; Panahi et al., 2019). The pathogenicity of variants in candidate genes with MAF ≤ 0.01 in all of the variant databases, including gnomAD, ExAC, and 1,000 Genomes, was compared with that of variants identified by Sanger sequencing.

### Corneal Examinations

In case that a candidate variant was identified in a family, the first-degree relatives of the patient were examined by slit-lamp, Pentacam rotating Scheimpflug tomography (software version 1.19r11; Oculus, Wetzlar, Germany), and other routine ophthalmological examinations. Multiple images and parameters were collected from the Pentacam data, based on corneal three-dimensional (3-D) tomography, including the “refractive maps” and the “Belin/Ambrosio Enhanced Ectasia Display (BAD)” maps, which had a good ability to detect the latent changes in early and subclinical stages (Ambrosio et al., 2011; Ruisenor Vazquez et al., 2014), and several parameters from BAD maps: thinnest pachymetry (TP), front and back elevation at the corneal thinnest location (F.Ele.Th and B.Ele.Th), pachymetric progression index average (PPIavg), Ambrosio relational thickness maximum (ARTmax), deviation of front elevation difference map and back elevation difference map (Df and Db), deviation of average pachymetric progression (Dp), deviation of minimum thickness (Dt), deviation of Ambrosio relational thickness maximum (Da), and total deviation value (BAD-D). All clinical data, including corneal signs by slit-lamp and images and parameters by 3-D tomography, were analyzed to compare phenotypic and subclinical phenotypic differences between KC patients and their first-degree relatives, especially the differences between the parents of the KC patient.

### Additional Data for Principal Component Analysis

For principal component analysis (PCA), additional 3-D tomography data of BAD maps were obtained from patients who were admitted to the Eye Hospital from November 2016 to October 2017. A total of 332 eyes in 187 patients with KC, 140 eyes in 70 patients with subclinical KC, and 490 eyes in 245 patients with myopia were enrolled. The myopic patients without other ocular diseases served as a control group. The subclinical group included the patients at high risk of developing KC or post-refractive surgery progressive corneal ectasia from refractive surgery candidates; they had at least three or more of the following conditions in at least one eye: (1) the total deviation parameter from BAD map (BAD-D) ≥ 1.6; (2) The back elevation at the corneal thinnest location parameter from BAD map (B.Ele.Th) ≥ 12; (3) abnormal (red) and/or suspicious (yellow) region(s) found in back elevation difference map from BAD map; (4) thinnest pachymetry < 510 mm; and (5) abnormal pattern in back elevation map from the “refractive maps,” such as island shape, tongue shape, or asymmetric hourglass shape. PCA was performed and visualized by Spyder (Version 2.3.8) with Python 2.7.

## Results

### Clinical Manifestations

In this study, all the 88 families included at least the proband and proband’s parents, and the number of participants in these families ranged from three to five. According to the preliminary diagnosis, 91 patients were diagnosed as having KC, including 88 probands in all families and three first-degree relatives in two Greek families (G1, G2). All the rest of first-degree relatives in 88 families were excluded from the diagnosis of clinical KC, without any self-reported complaints or symptoms.

### Genetic Variants in Patients With KC

**FIGURE 1:**
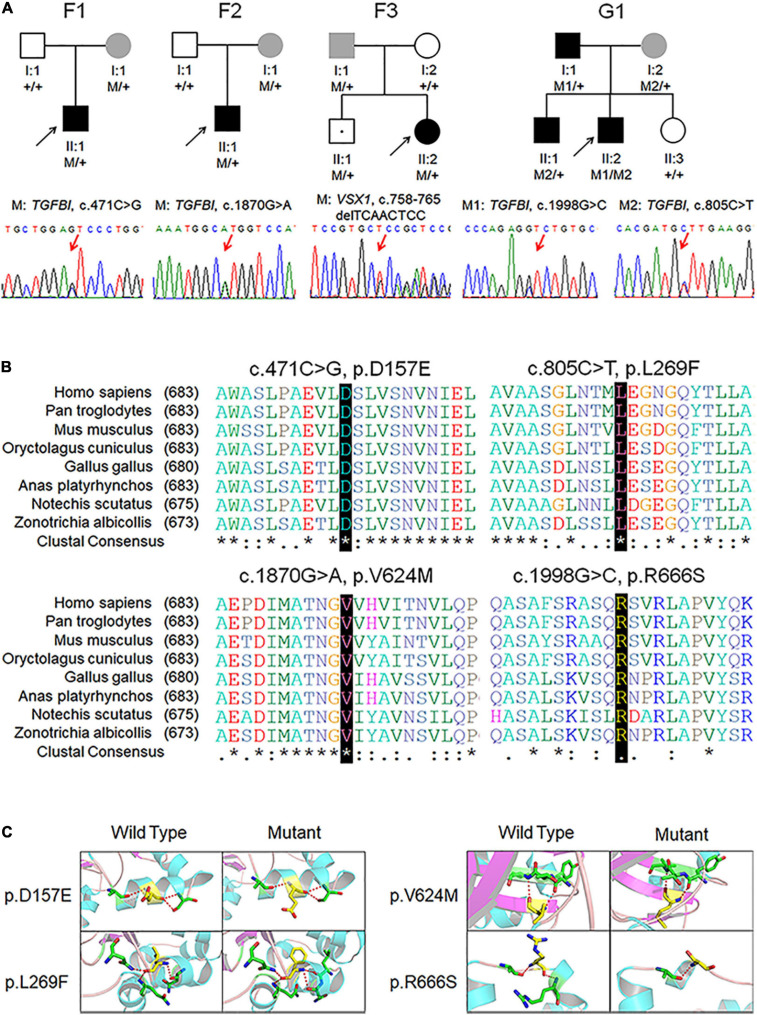
Pedigree, genotype, and clinical data of four probands with keratoconus. **(A)** Pedigree charts. Black symbol indicates affected member, white symbol indicates unaffected individual, gray symbol indicates member with subclinical keratoconus, and dotted symbol denotes variant carrier without any clinical feature. M represents a variant, and + indicates normal allele. The sequence chromatogram with red arrow represents mutant type. **(B)** Conservation analysis revealed evolutionary conservation of the variants. **(C)** Predicted three-dimensional structure of proteins.

Among the 88 probands with clinical KC, we identified five variants in *VSX1* and *TGFBI* genes. To summarize, a frameshift variant c.758-765delTCAACTCC (p.L253Rfs^∗^18) was found in *VSX1*, and 4 missense variants c.471C > G (p.D157E), c.805C > T (p.L269F), c.1870G > A (p.V624M) and c.1998G > C (p.R666S) were found in *TGFBI* (Table 1 and Figure 1A). Notably, c.805C > T (p.L269F) and c.1998G > C (p.R666S) in *TGFBI* were identified in a single Greek individual (family G1), and other variants were found in different patients from China (F1, F2, and F3).

**TABLE 1:** Bioinformatics data of variants identified by Sanger sequencing of the four probands.

Family	Source	Genotype	Gene	Amino acid change	1000 G	ExAC	gnomAD	Mutationtaster^*a*^	Polyphen2^*b*^	SIFT^*c*^
F1	Maternal	Het	*TGFBI*	c.471C > G:p.D157E	0.0008	0.00009	0.00012	D	P	T
F2	Maternal	Het	*TGFBI*	c.1870G > A:p.V624M	0.001	0.0003	0.00024	D	D	D
F3	Paternal	Het	*VSX1*	c.758-765delTCAACTCC p.L253Rfs*18	NI	0.00013	0.0002	D	NI	NI
G1	Maternal	Het	*TGFBI*	c.805C > T:p.L269F	0.0024	0.0018	0.00165	D	D	D
	Paternal	Het	*TGFBI*	c.1998G > C:p.R666S	0.0008	0.0016	0.00117	A	B	T

*Het, heterozygosis; NI, no information. ^*a*^MutationTaster: A, known to be deleterious; D, probably deleterious; N, probably harmless; P, known to be harmless. ^*b*^Polyphen2: D, probably damaging; P, possibly damaging; B, benign. ^*c*^SIFT: D, deleterious; T, tolerate.*

Identified variants were assessed by computational algorithms and human genome aggregation databases (Table 1). All variants were novel except variant c.1998G > C in *TGFBI*, which had been previously reported (Boutboul et al., 2006). All these variants were predicted deleterious, and the MAF of each one was less than 0.005 in gnomAD, ExAC, and 1000G. In addition, all missense variants in the *TGFBI* were highly conserved across different species (Figure 1B). Meanwhile, we predicted the three-dimensional structure of mutant proteins and wild-type ones for missense variants in *TGFBI*. Structure models demonstrated that D157E and L269F were located in a β-sheet and V624M in an α-helix, while L269F and R666S had a change in the hydrogen bonds (Figure 1C). In summary, we identified five variants in *VSX1* and *TGFBI* genes in four unrelated keratoconic families, four of which are reported for the first time.

### Whole-Exome Sequencing

To further verify the pathogenicity of the variants identified in *VSX1* and *TGFBI* genes, WES was performed in the four families (Supplementary Table 3). The mean depth of the WES targeted regions was > 60×, and the median coverage reached > 95%. As a result, WES captured the variants successfully, again confirming the variants in the *VSX1* and *TGFBI* genes. In addition, no better explainable variant was found in other known candidate genes responsible for KC, based on a comprehensive analysis of MAF, pathogenicity prediction, and co-segregation. In the absence of further functional studies, these data collectively indicate that the variants identified in both the patients and asymptomatic parents may be major genetic predisposing factors.

### Asymptomatic Relatives in the Families With Genetic Variants

We hypothesized that the relatives in the families are at high risk of developing KC even though they did not have self-reported symptoms. To test this hypothesis, we performed mutational screening in the four families with defined variants as mentioned above. Surprisingly, four asymptomatic relatives were found to carry disease-causing variants in an autosomal dominant inheritance pattern (Figure 1A).

In Chinese family F1, the asymptomatic mother (F1:I:2) who carried the missense variant c.471C > G in *TGFBI* gene transmitted it to the KC patient (F1:II:1). Clinical examination of the mother showed transverse diffused pigmentations in the central region of the cornea, and topographical examination showed five suspicious parameters (PPIavg, ARTmax, Dp, Da, and BAD-D) in the BAD maps of the right eye. Furthermore, there was a suspicious circular region highlighted in yellow in the “difference elevation map” of the front surface of cornea in the left eye with two suspicious parameters (Dt and BAD-D). The topographic parameters of the unaffected father (F1:I:1) were almost normal except a slightly abnormal BAD-D parameter in both eyes (Supplementary Figure 1 and Supplementary Table 4).

**FIGURE 2:**
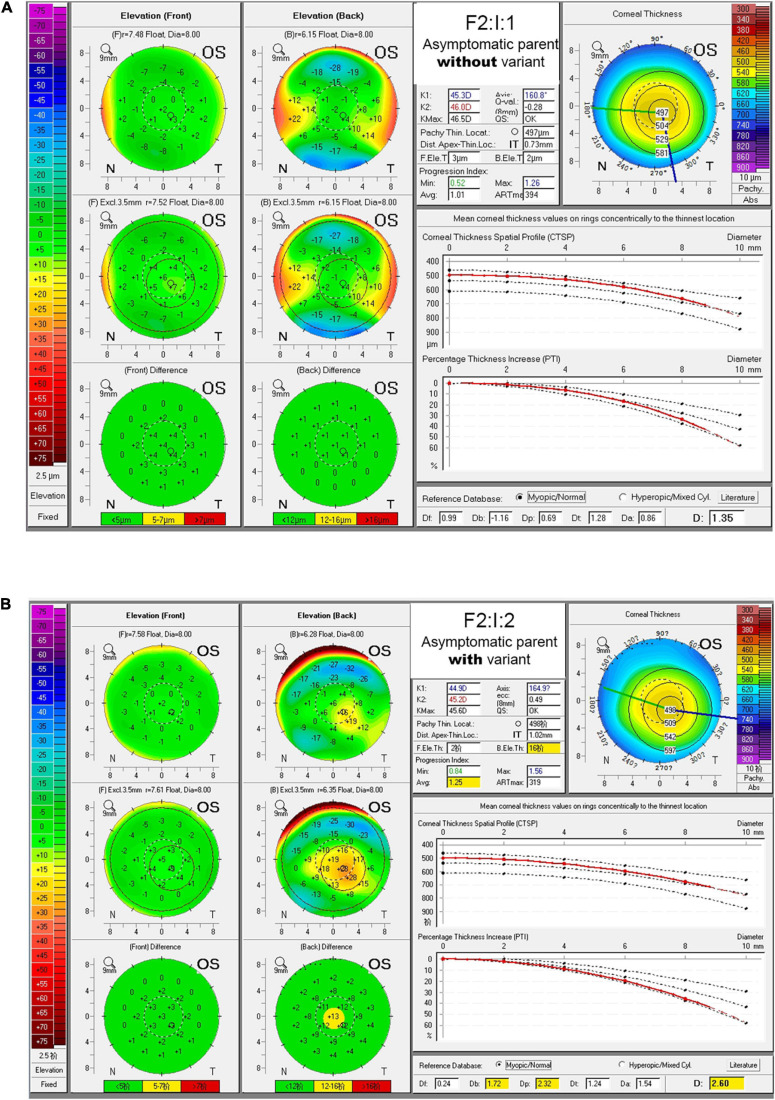
BAD maps of left eye of the asymptomatic parents with different genotypes in an example family. **(A)** Tomographic display from “Belin/Ambrosio Enhanced Ectasia Display (BAD)” maps of the asymptomatic parent without variant (F2:I:1). **(B)** Tomographic display from BAD maps of the asymptomatic parent with variant (F2:I:2). Region highlighted in yellow in the “difference elevation map” indicates suspicious region. OD, right eye; OS, left eye.

In Chinese family F2, the *TGFBI* variant c.1870G > A was identified in both the KC patient (F2:II:1) and the mother (F2:I:2) who had no self-conscious symptom. The mother was found to have dour suspicious parameters (B.Ele.Th, PPIavg, Dp, and BAD-D) in BAD maps of the right eye, while a yellow region in the back “difference elevation map” with five suspicious parameters (B.Ele.Th, PPIavg, Db, Dp, and BAD-D) was found in the left eye. The father (F2: I:1) was unaffected with KC but had an explicit history of trauma and pterygium in the right eye (Figure 2, Supplementary Figure 2, and Supplementary Table 4).

In the third Chinese family (F3), two siblings (F3:II:2 and F3:II:1) carried the frameshift variant c.758-765delTCAACTCC in *VSX1* gene which was inferably inherited from the asymptomatic father (F3:I:1) who was found to have transverse diffused pigmentations in the inferior region of the cornea in both eyes and a suspicious region in the back “difference elevation map” in the right eye. The father was found to have three suspicious parameters (ARTmax, Da, and BAD-D) and three abnormal parameters (B.Ele.Th, PPIavg, and Dp) in BAD maps of the right eye, while three suspicious parameters (PPIavg, Dp, and BAD-D) were found in the left eye. In the mother (F3:I:2), only a slightly suspicious BAD-D value (1.61) was found in the left eye. The brother (F3:II:1) also had no suspicious clinical finding (Supplementary Figure 3 and Supplementary Table 4).

In the Greek family (G1), the proband (G1:II:2) carried two different heterozygous variants in the same gene *TGFBI*. The variant c.1998G > C was inherited from the affected father (G1:I:1) who had received cross-linking therapy in both eyes, while the variant c.805C > T was shared with the affected brother (G1:II:1), and both inherited from the asymptomatic mother (G1:I:2) who was found to have three suspicious parameters (TP, Dt, and BAD-D) in BAD maps of the right eye and four suspicious parameters (TP, B.Ele.Th, Dt, and BAD-D) in the left eye. The sister (G1:II:3), without detectable variant, had no suspicious clinical finding. In addition, compared to the father and brother, the proband had more severe symptoms and earlier onset age of the KC (Supplementary Figure 4 and Supplementary Table 4).

**FIGURE 3:**
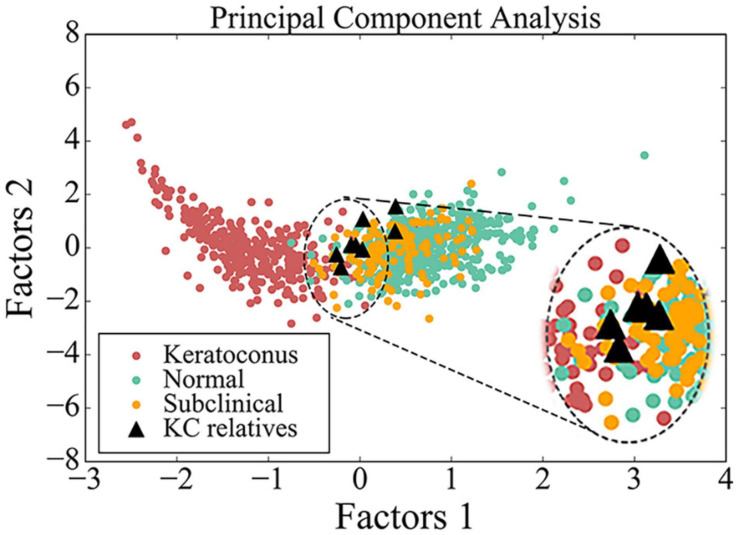
Scatter diagram of data from BAD maps in different groups by principal component analysis. Red circle indicates data from patient with keratoconus, green circle indicates data from control, yellow circle indicates data from subclinical one, and black triangle indicates data from the asymptomatic parent with variant in this study. The image in the upper right corner is a larger image of the dotted line area.

PCA had been employed to compare corneal parameters in BAD maps of the KC group, subclinical group, myopia group, and eight eyes in four subclinical parents in this study (Figure 3). We observed that most eyes (6/8) from the four subclinical parents just happen to be in the border region (dotted line area) between myopia group and KC group.

**FIGURE 4:**
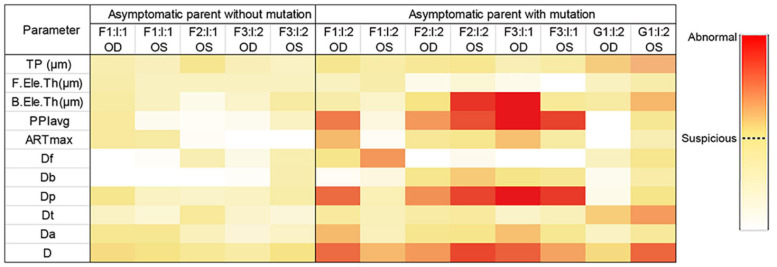
Heatmap of parameters from Pentacam of the parents in the four families. Data visualization was carried out based on the different value range of each parameter, and the darker color represents the higher probability of anomaly. *Data of the right eye of F2: I:1 was excluded from our study because of pterygium, corneal macula, and explicit history of trauma. OD, right eye; OS, left eye.

Taken together, genetic variants together with ultra-early corneal topographical changes were found in asymptomatic parents (Figure 4), supporting that the relatives in KC families are of higher risk of developing a latent subclinical KC.

## Discussion

Patients with KC often exhibit a range of disease manifestations of varying severity (Rabinowitz, 1998). Importantly, post-refractive surgery progressive corneal ectasia had occurred in many patients who did not exhibit any clinical sign of risk factors prior to surgery (Klein et al., 2006). Based on the fact that KC has a clear genetic tendency, previous studies have found a large number of families with dominant inheritance (Paliwal et al., 2011). Given the genetic basis of this disease, we screened the genetic variants in two cohorts from China and Greek. With the identified variants in KC patients by Sanger sequencing and WES, we found the same variants in the asymptomatic parents in these families. Further corneal topographical examination discovered the subclinical abnormalities in these parents.

Most patients with KC were sporadic in previous reports (Romero-Jimenez et al., 2010); however, familial aggregation of KC and high prevalence of first-degree relatives of patients with KC had been widely reported. In addition, several scholars had conducted in-depth studies on the corneal topography of first-degree relatives. Awwad et al. (2019) recruited 183 first-degree relatives aged 6 to 18 years of patients with KC for clinical research. Finally, 32 relatives (17.5%) were revealed as having KC by tomographic evaluation, and the unilateral KC accounted for 37.5%. They concluded that first-degree relatives of KC patients were at high risk of developing KC, and the high rate of unilateral KC suggested a high likelihood of developing KC from normal eyes of first-degree relatives. Kaya et al. (2008) analyzed the corneal topographic features of 72 first-degree relatives of patients with KC and found that eight of them were diagnosed as having KC. Meanwhile, in the remaining 64 asymptomatic relatives, more abnormal corneal parameters could be detected, compared with the control group. Shneor et al. (2020). also found that 18% of first-degree relatives were diagnosed with KC or KC suspect, and 34% had at least one abnormal corneal parameter. Steele et al. (2008) found that KC traits were more likely to appear in the corneal topography of first-degree relatives of patients with KC and suggested an autosomal dominant pattern of inheritance with variable expressivity in some families. Karimian et al. (2008). found that relatives of patients with KC had high incidence of latent KC through topographic evaluation. In our study, we found four parents without self-reported symptoms indeed carry genetic variants in the *VSX1* and *TGFBI* genes. Furthermore, corneal examinations revealed subclinical changes in the cornea. These findings are suggestive of disease predisposition by the same genetic variants with various degrees of disease expressivity and, more importantly, warrant further study on asymptomatic family members and early diagnosis of KC. We speculate that the asymptomatic family members of the KC patients are high-risk individuals based on the significant genetic trend of this disease and further hypothesize that there are many similarly high-risk individuals in the population.

From PCA diagram (Figure 3), we can observe the continuity between the patient group and the control group, which is consistent with the clinical situation, because there is no clear boundary between KC patients and normal people. In addition, most eyes of the four subclinical parents (6/8) just happen to be in the critical region (dotted line area) between the two groups, as well as many from the subclinical population, so we speculate that many people similar to them may be at potential risk.

With the sharp increase of myopic population globally, there is a huge demand for refractive surgery, which makes a large number of high-risk individuals face the risk of postoperative corneal ectasia after refractive surgery. Post-refractive surgery progressive corneal ectasia is a rare but severe complication of refractive surgery. In the absence of recognized classification system for KC, especially for the subclinical KC, it is important to find a way to accurately screen these high-risk groups. However, despite the rapid development of corneal measurement technology, it is still hard to find a clinical approach with sufficient sensitivity and specificity to identify subclinical KC. Our study supports that genetic screening is important in detecting subclinical KC in extremely early stage or even suspects without any clinical signs.

In conclusion, we performed genetic screening in a large cohort of Chinese and Greek patients with KC and suggested that variants in the *VSX1* and *TGFBI* genes might be responsible for KC through autosomal dominant inheritance pattern with variable expressivity. Further functional studies will be conducted in the future to verify the results of this study. At the same time, the asymptomatic members in keratoconic families were at high risk to carry subclinical KC and should be encouraged to undertake molecular screening and Pentacam Scheimpflug tomography. Our finding suggested that “Belin/Ambrosio Enhanced Ectasia Display” on the Pentacam plays an important role in detecting the latent subclinical changes, and genetic screening is of great value in establishing disease classification system of subclinical and early-stage KC and for the preoperative screening of refractive surgery individuals to prevent postoperative corneal ectasia.

## Data Availability Statement

The data presented in the study are deposited in the Figshare repository. It can be found here: https://figshare.com/s/3658e55605f0f001ca74 (doi: 10.6084/m9.figshare.14378519).

## Ethics Statement

The studies involving human participants were reviewed and approved by Institutional Review Board of The Eye Hospital of Wenzhou Medical University and the Emmetropia Mediterranean Eye Institute. Written informed consent to participate in this study was provided by the participants’ legal guardian/next of kin.

## Author Contributions

Z-BJ designed the experiments and supervised the whole study. SC, X-YL, J-JJ, Z-JC, J-YM, R-JS, EL, SV, and IA performed the experiments. SC, X-YL, F-FC, and IA analyzed the data. SC and X-YL wrote the manuscript. All authors contributed to manuscript revision, read, and approved the submitted version.

## Conflict of Interest

The authors declare that the research was conducted in the absence of any commercial or financial relationships that could be construed as a potential conflict of interest.

## Footnotes

1 http://mutationtaster.org/

2 http://genetics.bwh.harvard.edu/pph2/

3 http://sift.jcvi.org/www/SIFT_enst_submit.html

4 http://www.ncbi.nlm.nih.gov/protein

5 http://www.sbg.bio.ic.ac.uk/servers/phyre2/html/page.cgi?id=index

## Abbreviations

ARTmax: Ambrosio relational thickness maximum
BAD: Belin/Ambrosio enhanced ectasia display
BAD-D: total deviation value of Belin/Ambrosio enhanced ectasia display
B.Ele.Th: back elevation at the corneal thinnest location
Da: deviation of Ambrosio relational thickness maximum
Db: deviation of back elevation difference map
Df: deviation of front elevation difference map
Dp: deviation of average pachymetric progression
Dt: deviation of minimum thickness
ExAC: exome aggregation consortium
F.Ele.Th: front elevation at the corneal thinnest location
gnomAD: genome aggregation database
KC: keratoconus
MAF: minor allele frequency
PCA: principal component analysis
PPIavg: pachymetric progression index average
TP: thinnest pachymetry
WES: whole-exome sequencing
1000G: 1,000 genome.
