# Supplementary material for: Genetic Screening Revealed Latent Keratoconus in Asymptomatic Individuals
Source: Front Cell Dev Biol. 2021 May 31;9:650344. doi: 10.3389/fcell.2021.650344 (PMC8202288; doi:10.3389/fcell.2021.650344)
Supplement: Supplementary file 1 [file Data_Sheet_1.PDF]

Supplementary Material

1 Supplementary Figures and Tables

1.1 Supplementary Figures

A

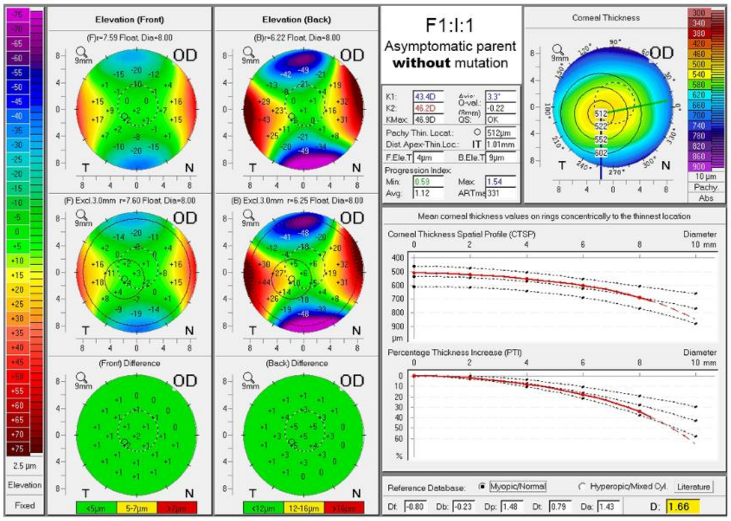

B

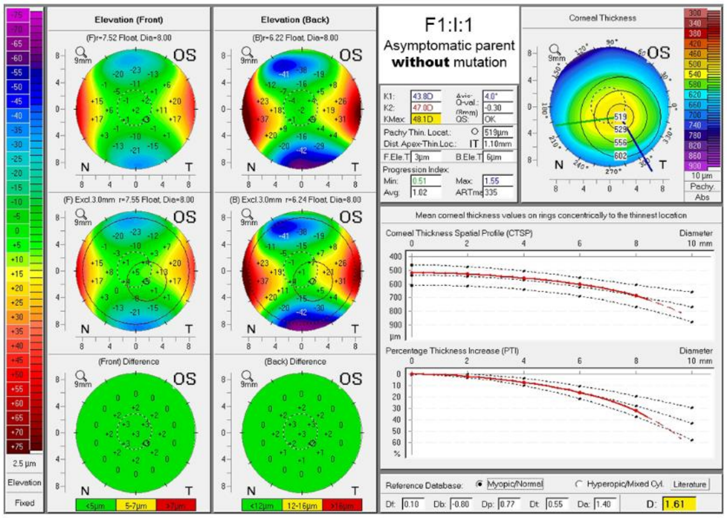

Supplementary Figure 1. BAD maps of first-degree relatives in family F1. (A) (B) 'Belin-Ambrosio Enhanced Ectasia Display (BAD)' maps of the asymptomatic parent without mutation (F1:I:1). **(C) (D)** BAD maps of the asymptomatic parent with mutation (F1:I:2). Region highlighted in *yellow* in the 'difference elevation map' indicates suspicious region. Parameter in *yellow* indicates suspicious parameter, parameter in *red* indicates abnormal parameter. OD=Right eye. OS=Left eye.

C

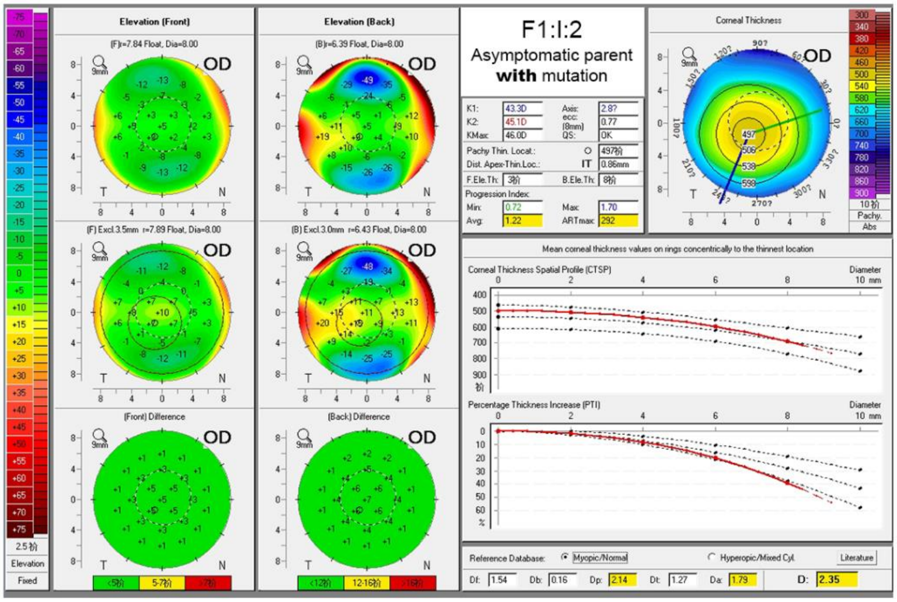

D

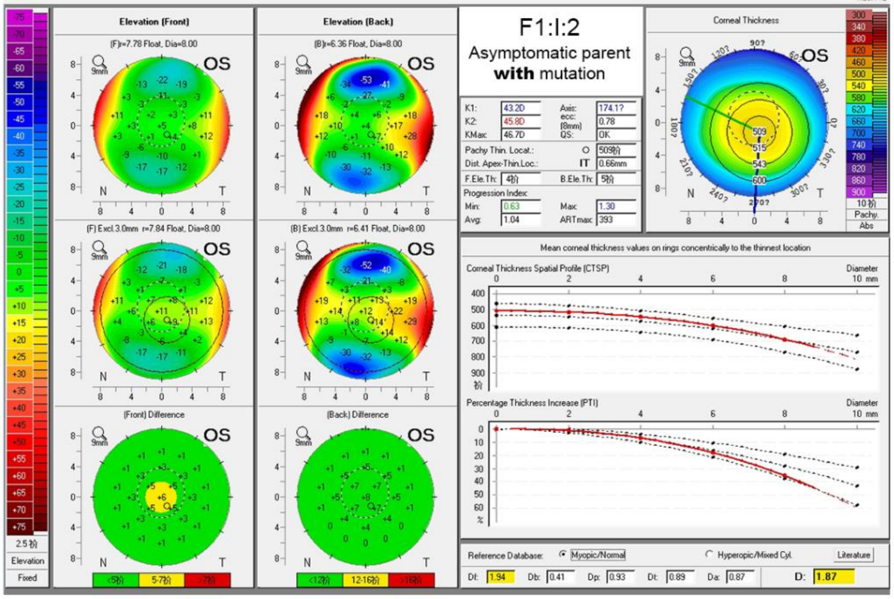

Supplementary Figure 2. BAD maps of first-degree relatives in family F1. (A) (B) ‘Belin-Ambrosio Enhanced Ectasia Display (BAD)’ maps of the asymptomatic parent without mutation (F1:I:1). **(C) (D)** BAD maps of the asymptomatic parent with mutation (F1:I:2). Region highlighted in *yellow* in the ‘difference elevation map’ indicates suspicious region. Parameter in *yellow* indicates suspicious parameter, parameter in *red* indicates abnormal parameter. OD=Right eye. OS=Left eye.

A

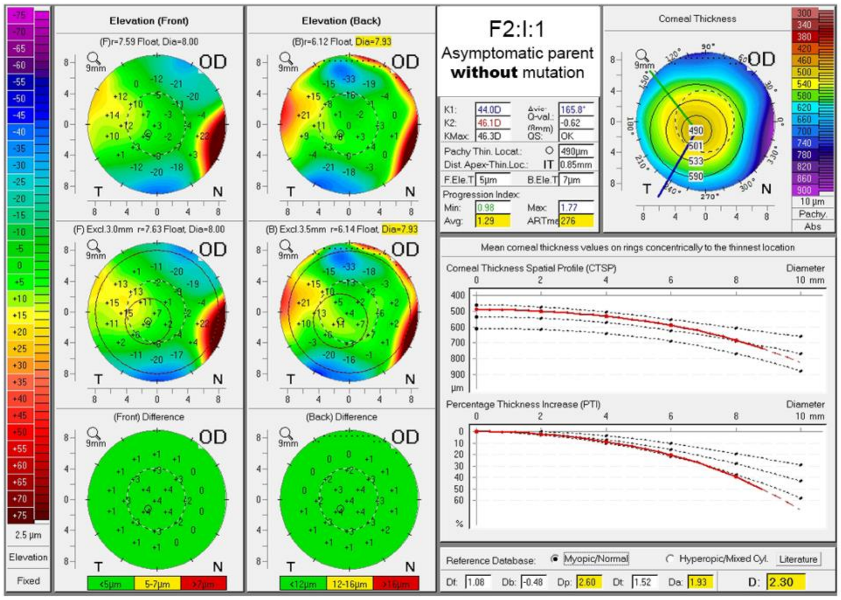

B

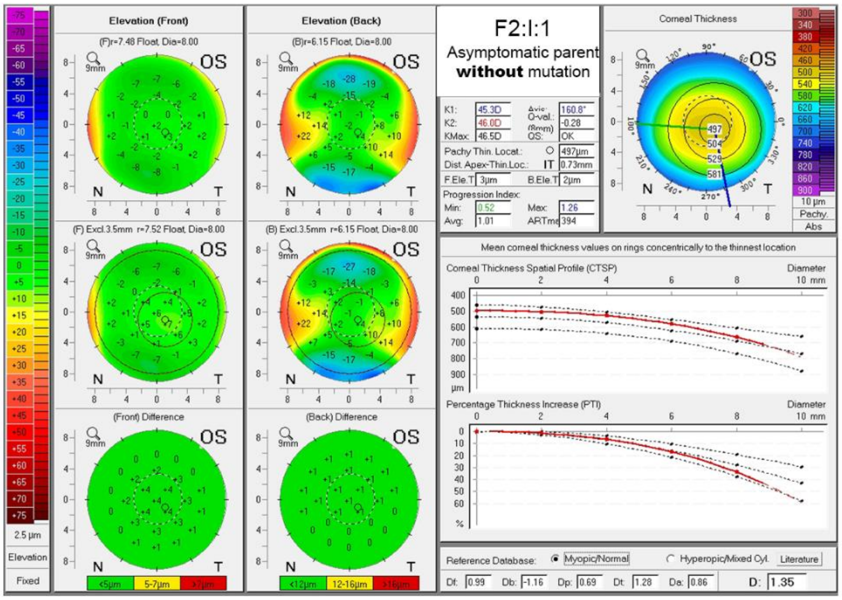

Supplementary Figure 2. BAD maps of first-degree relatives in family F2. (A) (B) 'Belin-Ambrosio Enhanced Ectasia Display (BAD)' maps of the asymptomatic parent without mutation (F2:I:1*). **(C) (D)** BAD maps of the asymptomatic parent with mutation (F2:I:2). Region highlighted in *yellow* in the 'difference elevation map' indicates suspicious region. Parameter in yellow indicates suspicious parameter, parameter in *red* indicates abnormal parameter. OD=Right eye. OS=Left eye. *The right eye of F2: I:1 had pterygium, corneal macula, and explicit history of trauma, so the corneal data of right eye was excluded from our study.

C

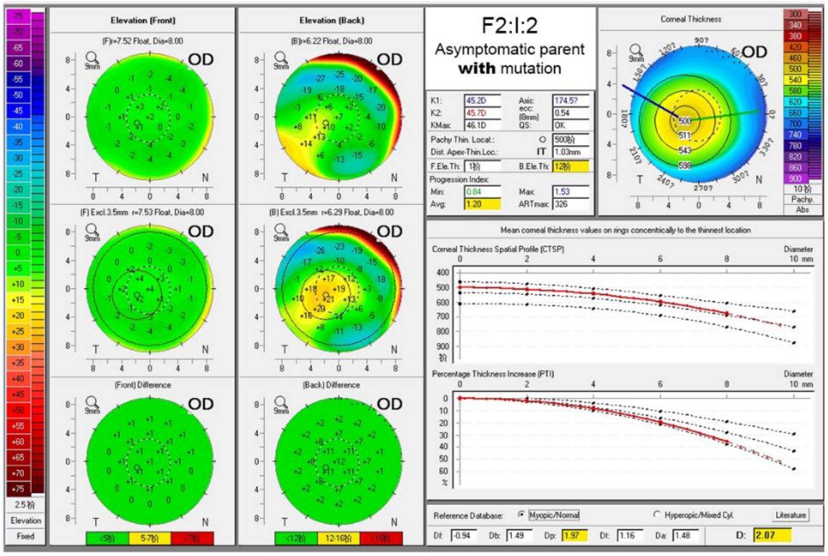

D

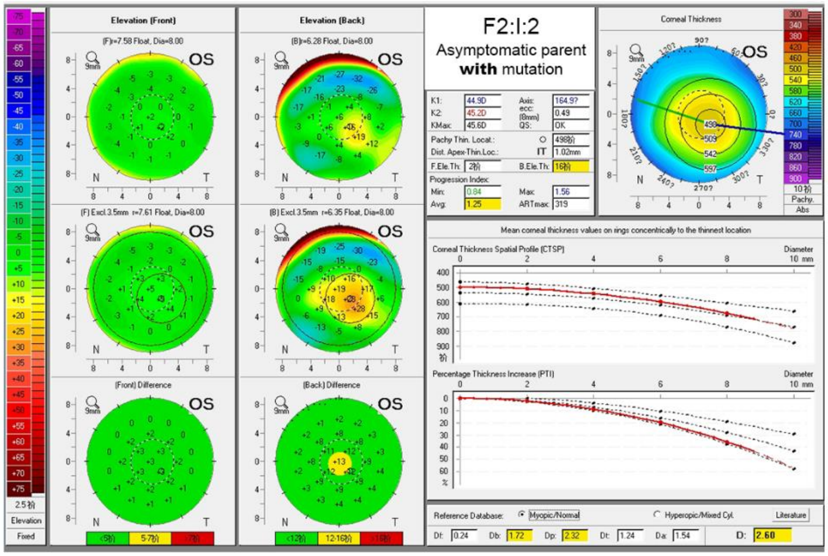

Supplementary Figure 2. BAD maps of first-degree relatives in family F2. (A) (B) 'Belin-Ambrosio Enhanced Ectasia Display (BAD)' maps of the asymptomatic parent without mutation (F2:I:1*). **(C) (D)** BAD maps of the asymptomatic parent with mutation (F2:I:2). Region highlighted in *yellow* in the 'difference elevation map' indicates suspicious region. Parameter in yellow indicates suspicious parameter, parameter in *red* indicates abnormal parameter. OD=Right eye. OS=Left eye. *The right eye of F2: I:1 had pterygium, corneal macula, and explicit history of trauma, so the corneal data of right eye was excluded from our study.

A

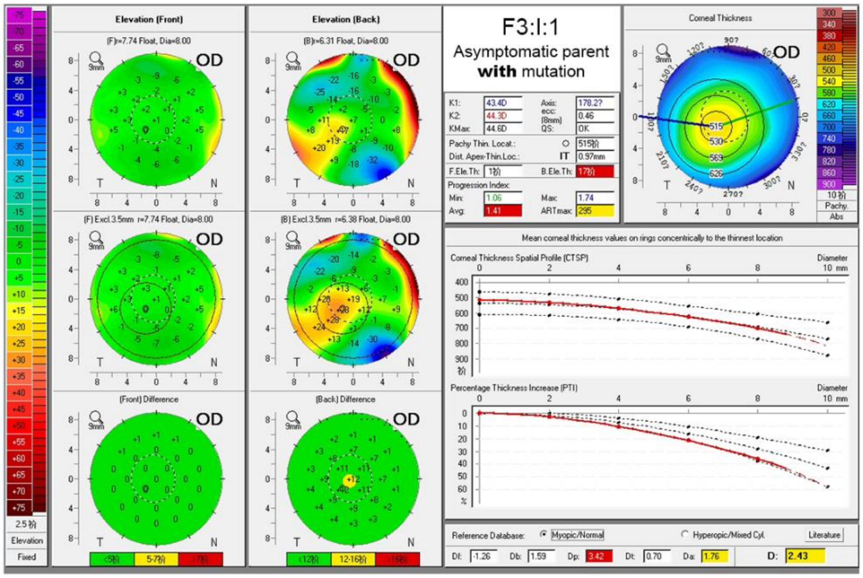

B

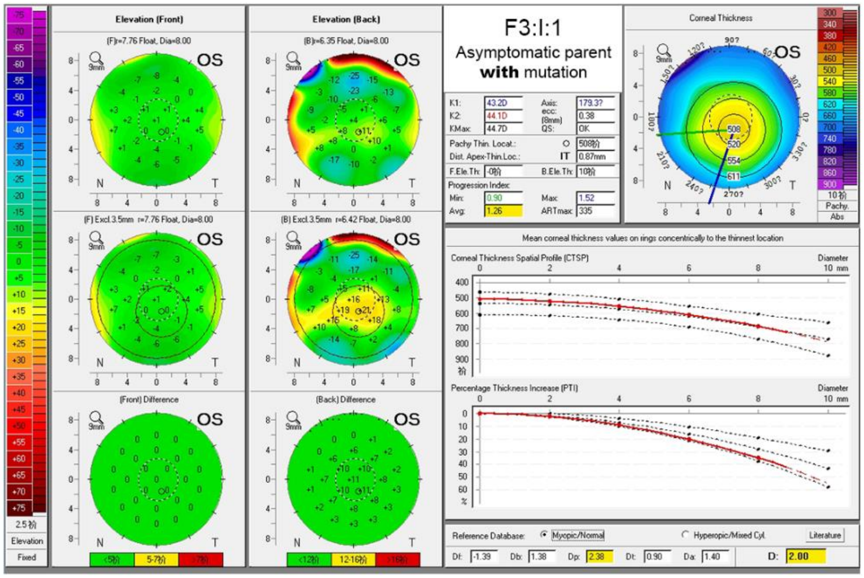

Supplementary Figure 3. BAD maps of first-degree relatives in family F3. (A) (B) ‘Belin-Ambrosio Enhanced Ectasia Display (BAD)’ maps of the asymptomatic parent with mutation (F3:I:1). **(C) (D)** BAD maps of the asymptomatic parent without mutation (F3:I:2). **(E) (F)** BAD maps of the asymptomatic sibling with mutation (F3:II:1). Region highlighted in *yellow* in the ‘difference elevation map’ indicates suspicious region. Parameter in *yellow* indicates suspicious parameter, parameter in *red* indicates abnormal parameter. OD=Right eye. OS=Left eye.

C

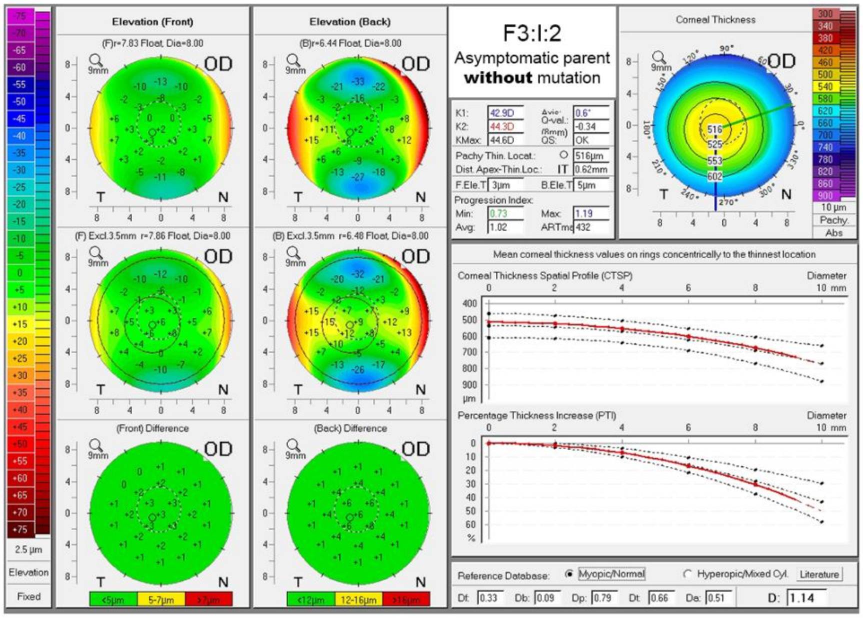

D

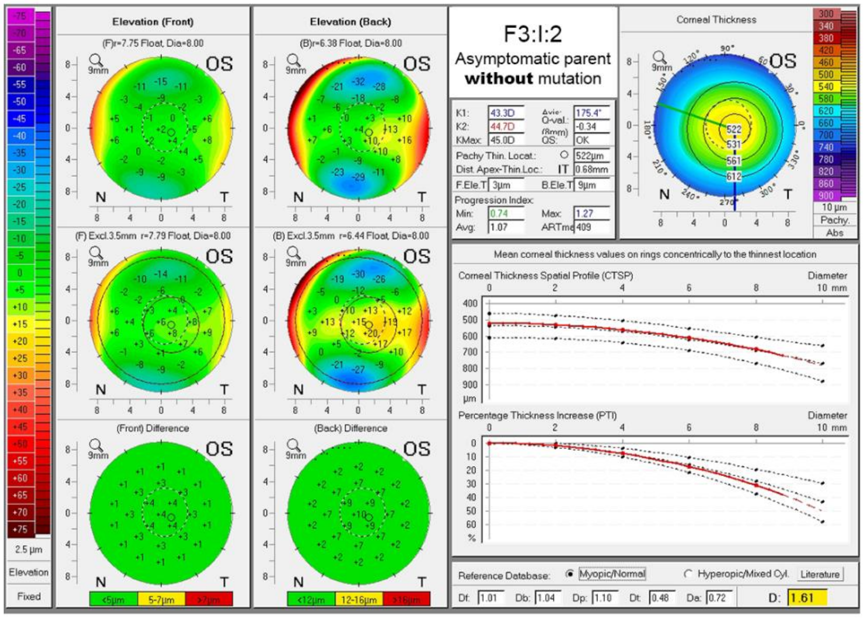

Supplementary Figure 3. BAD maps of first-degree relatives in family F3. (A) (B) 'Belin-Ambrosio Enhanced Ectasia Display (BAD)' maps of the asymptomatic parent with mutation (F3:I:1). **(C) (D)** BAD maps of the asymptomatic parent without mutation (F3:I:2). **(E) (F)** BAD maps of the asymptomatic sibling with mutation (F3:II:1). Region highlighted in *yellow* in the 'difference elevation map' indicates suspicious region. Parameter in *yellow* indicates suspicious parameter, parameter in *red* indicates abnormal parameter. OD=Right eye. OS=Left eye.

E

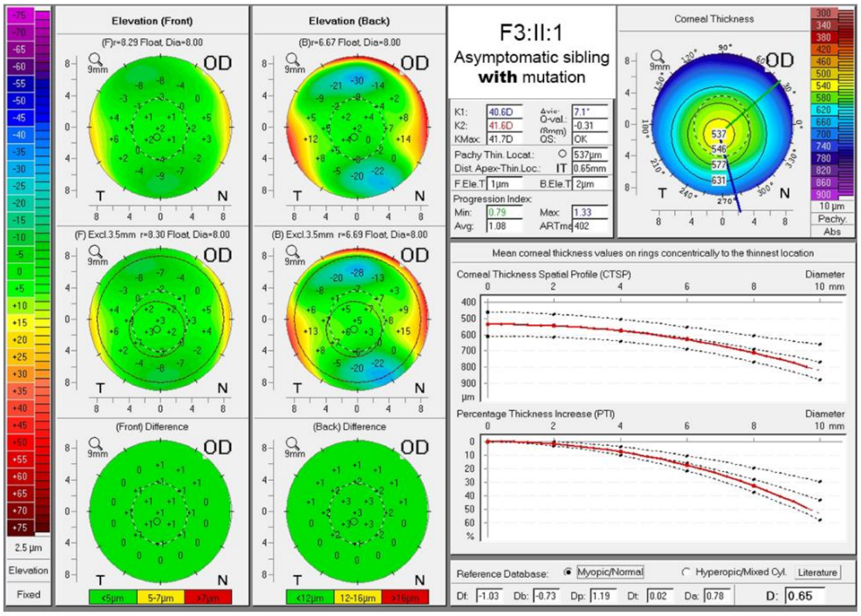

F

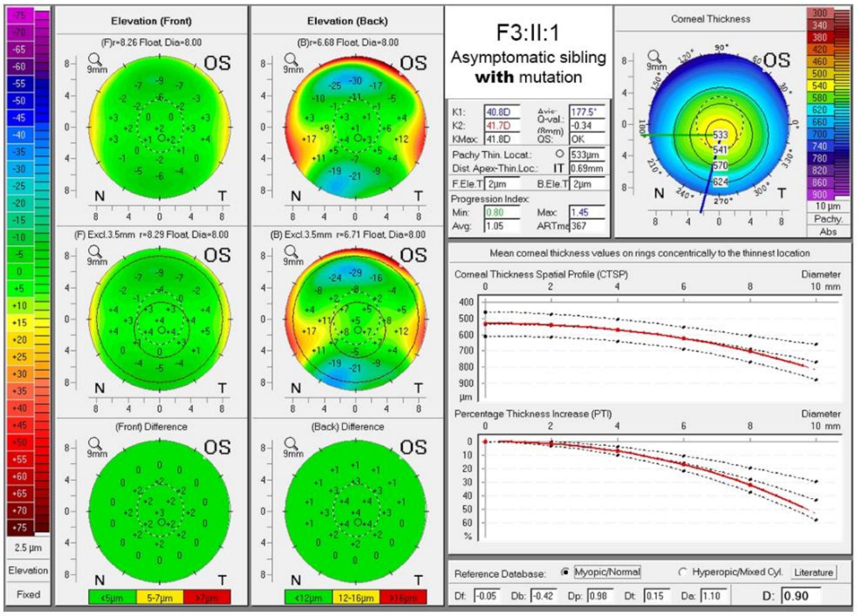

Supplementary Figure 3. BAD maps of first-degree relatives in family F3. (A) (B) 'Belin-Ambrosio Enhanced Ectasia Display (BAD)' maps of the asymptomatic parent with mutation (F3:I:1). **(C) (D)** BAD maps of the asymptomatic parent without mutation (F3:I:2). **(E) (F)** BAD maps of the asymptomatic sibling with mutation (F3:II:1). Region highlighted in *yellow* in the 'difference elevation map' indicates suspicious region. Parameter in *yellow* indicates suspicious parameter, parameter in *red* indicates abnormal parameter. OD=Right eye. OS=Left eye.

A

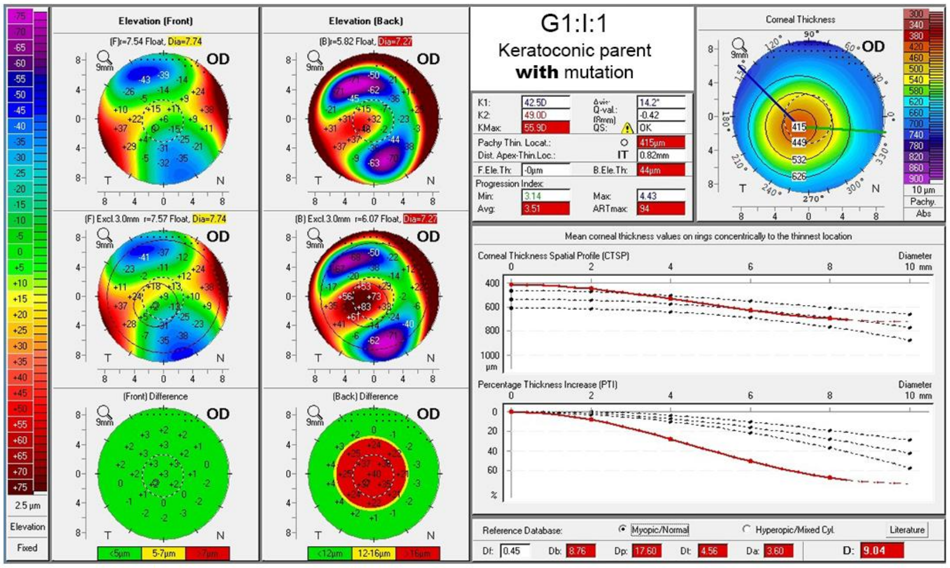

B

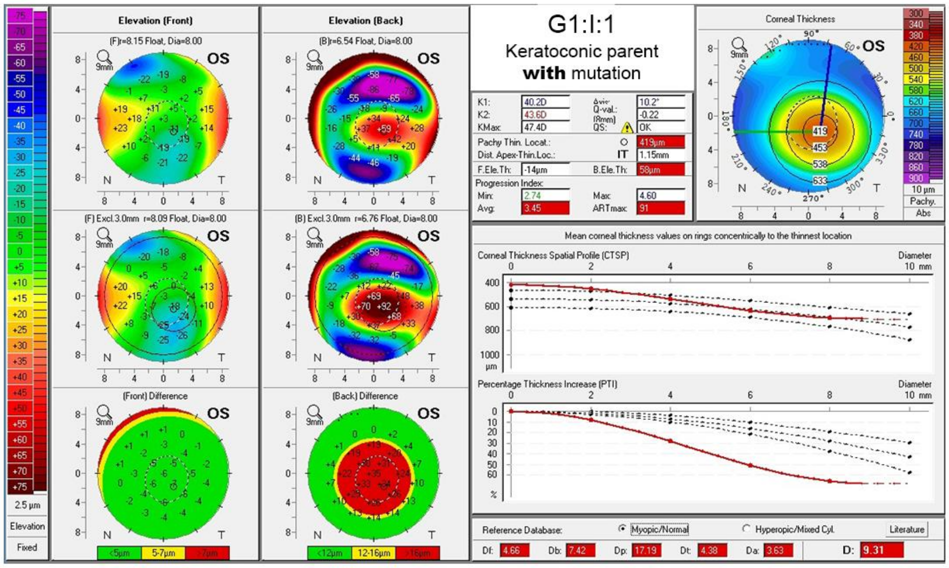

Supplementary Figure 4. BAD maps of first-degree relatives in family G1. (A) (B) ‘Belin-Ambrosio Enhanced Ectasia Display (BAD)’ maps of the keratoconic parent with mutation (G1:I:1). **(C) (D)** BAD maps of the asymptomatic parent with mutation (G1:I:2). **(E) (F)** BAD maps of the keratoconic sibling with mutation (G1:II:1). **(G) (H)** BAD maps of the asymptomatic sibling without mutation (G1:II:3). Region highlighted in *yellow* in the ‘difference elevation map’ indicates suspicious region. Parameter in *yellow* indicates suspicious parameter, parameter in *red* indicates abnormal parameter. OD=Right eye. OS=Left eye.

C

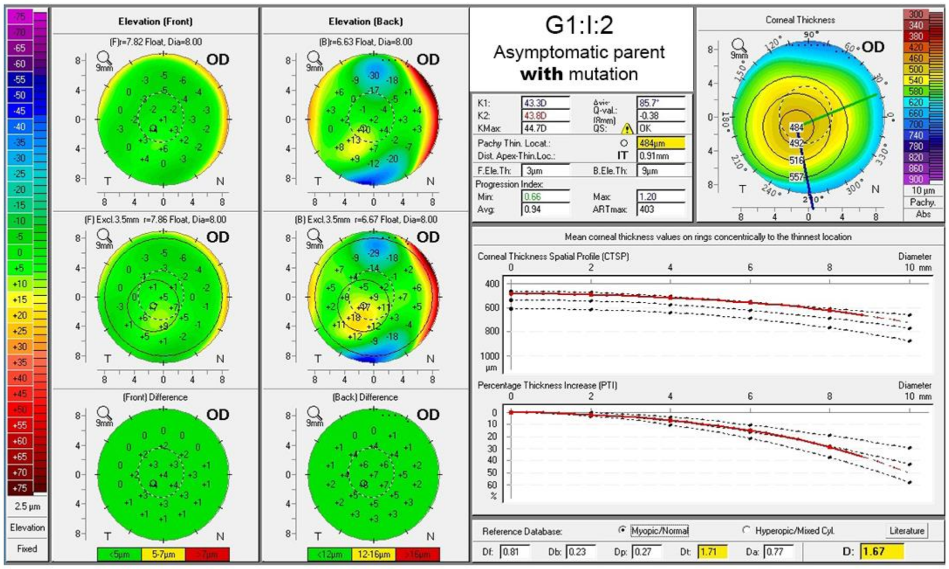

D

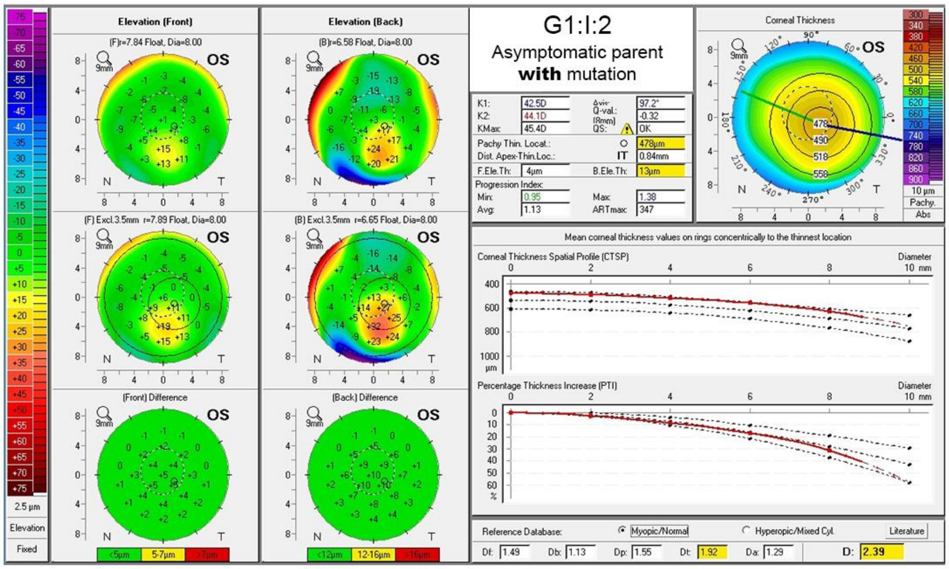

Supplementary Figure 4. BAD maps of first-degree relatives in family G1. (A) (B) 'Belin-Ambrosio Enhanced Ectasia Display (BAD)' maps of the keratoconic parent with mutation (G1:I:1). **(C) (D)** BAD maps of the asymptomatic parent with mutation (G1:I:2). **(E) (F)** BAD maps of the keratoconic sibling with mutation (G1:II:1). **(G) (H)** BAD maps of the asymptomatic sibling without mutation (G1:II:3). Region highlighted in *yellow* in the 'difference elevation map' indicates suspicious region. Parameter in *yellow* indicates suspicious parameter, parameter in *red* indicates abnormal parameter. OD=Right eye. OS=Left eye.

E

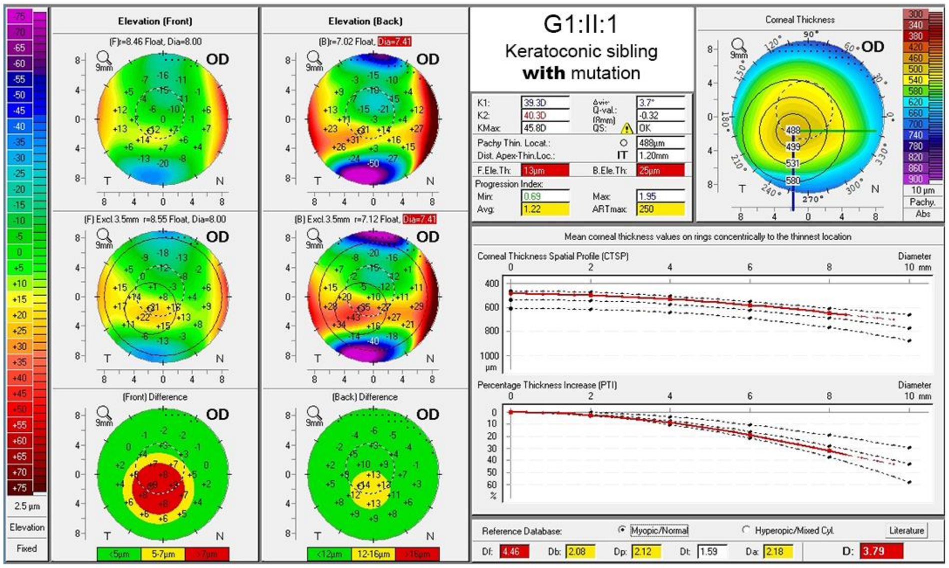

F

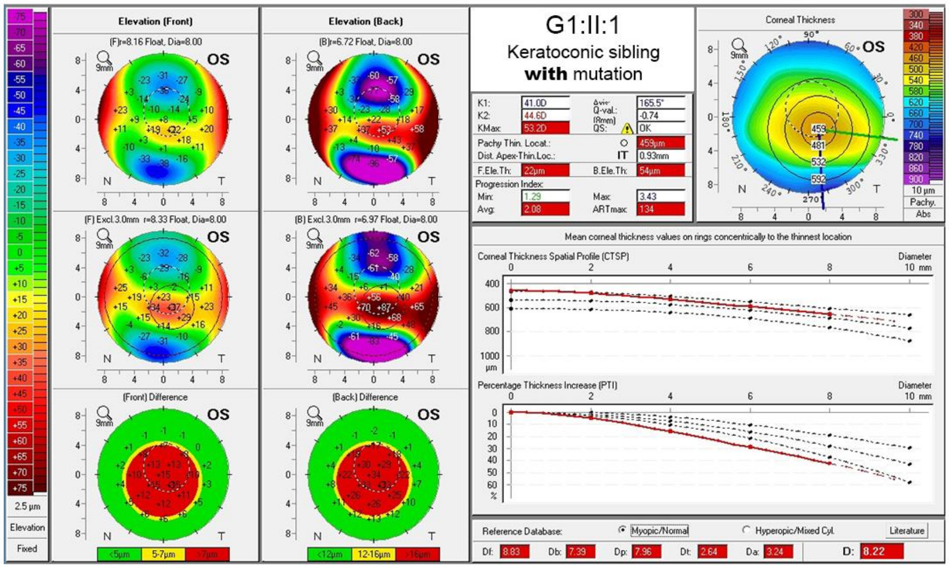

Supplementary Figure 4. BAD maps of first-degree relatives in family G1. (A) (B) ‘Belin-Ambrosio Enhanced Ectasia Display (BAD)’ maps of the keratoconic parent with mutation (G1:I:1). **(C) (D)** BAD maps of the asymptomatic parent with mutation (G1:I:2). **(E) (F)** BAD maps of the keratoconic sibling with mutation (G1:II:1). **(G) (H)** BAD maps of the asymptomatic sibling without mutation (G1:II:3). Region highlighted in *yellow* in the ‘difference elevation map’ indicates suspicious region. Parameter in *yellow* indicates suspicious parameter, parameter in *red* indicates abnormal parameter. OD=Right eye. OS=Left eye.

G

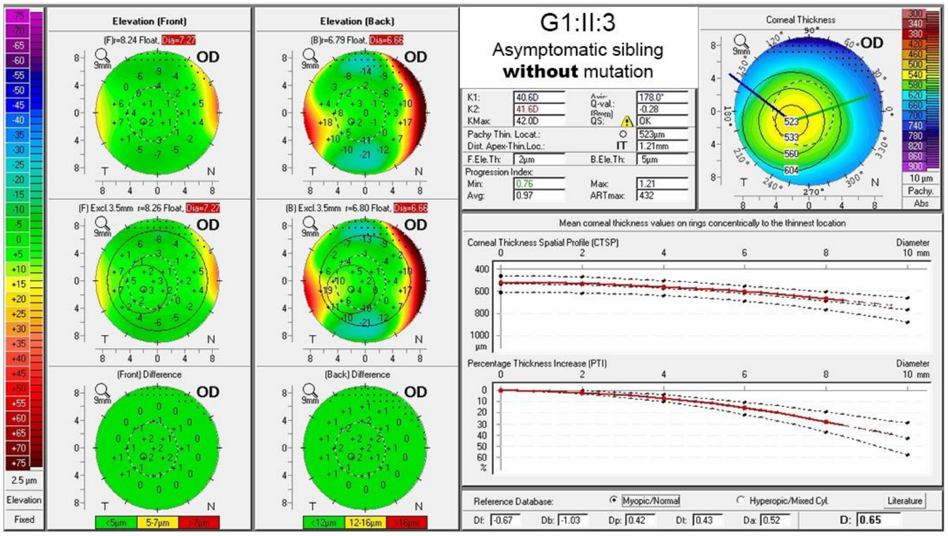

H

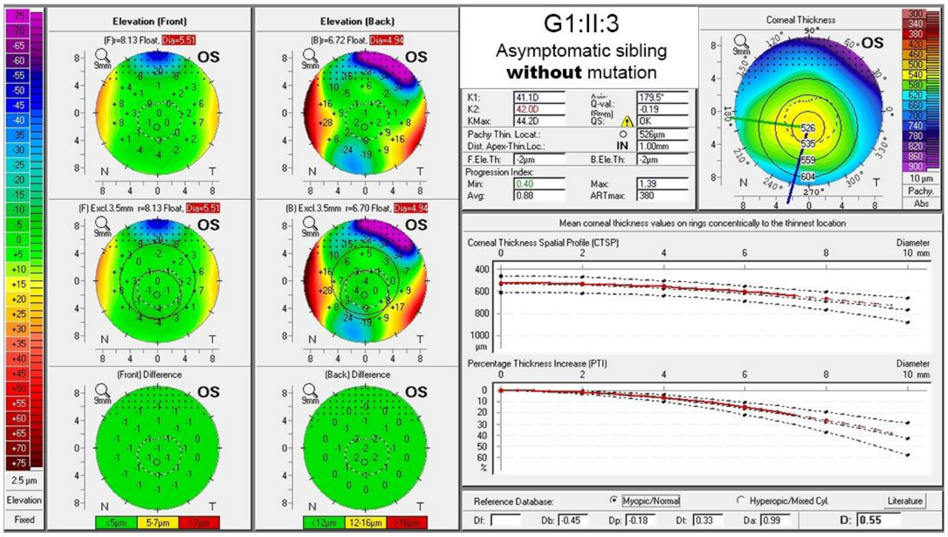

Supplementary Figure 4. BAD maps of first-degree relatives in family G1. (A) (B) ‘Belin-Ambrosio Enhanced Ectasia Display (BAD)’ maps of the keratoconic parent with mutation (G1:I:1). **(C) (D)** BAD maps of the asymptomatic parent with mutation (G1:I:2). **(E) (F)** BAD maps of the keratoconic sibling with mutation (G1:II:1). **(G) (H)** BAD maps of the asymptomatic sibling without mutation (G1:II:3). Region highlighted in *yellow* in the ‘difference elevation map’ indicates suspicious region. Parameter in *yellow* indicates suspicious parameter, parameter in *red* indicates abnormal parameter. OD=Right eye. OS=Left eye.

1.2 Supplementary Tables

Supplementary Table 1. Primers for *VSX1*, *TGFBI* and *ZEB1* amplification.

Primer[a]	Sequence	TM(°C)	Product size(bp)
VSX1_1F	5’-CACGCCAGGATGTAGAACTT-3’	58	869
VSX1_1R	5’-GGGCGATGGTCTGTGAC-3’		
VSX1_2F	5’-CCTGGGATCTAGTAGGCACTAA-3’	60	328
VSX1_2R	5’-CTATCATGCCGGGCCATAAA-3’		
VSX1_3F	5’-TAGGGTCTGGACAGCAGAG-3’	58	563
VSX1_3R	5’-CCTCAGGCATTTGTGTTGATTT-3’		
VSX1_4F	5’-GTGTCCCTACTGCGTTGAAT-3’	58	525
VSX1_4R	5’-ACTGACGTTGCTTTGCTTTG-3’		
VSX1_5F	5’-CAATGCCAATCACTGTGTCATC-3’	58	691
VSX1_5R	5’-AGGTCACTCATCTGTCCTCTTA-3’		
VSX1_6F	5’-GGCAGCATCTCAGGACTT-3’	57	412
VSX1_6R	5’-TGCTGCCTGCTCCTATTC-3’		
VSX1_7F	5’-GTTCACTAAGCCACAGTCTCTC-3’	58	581
VSX1_7R	5’-CTGGTATGGACAGTTCCCTTTC-3’		
TGFBI_1F	5’-TGGTTTGAGGAAGACTGTGG-3’	60	582
TGFBI_1R	5’-GCTCCATGCTGCAAGGTTT-3’		
TGFBI_2F	5’-TAGAAAGTTCCAGTGACCCAAG-3’	60	317
TGFBI_2R	5’-CACATGCATCTTCATGGCTTT-3’		
TGFBI_3F	5’-CTCAGGAAAGGCAGACCTATG-3’	60	439
TGFBI_3R	5’-GAGGAGCAGCTCAGGAAATG-3’		
TGFBI_4F	5’-ATGTCTGGGAACAAGAGCTG-3’	58	536
TGFBI_4R	5’-GTAGGAAATGACTGCAGACTCC-3’		
TGFBI_5F	5’-TGCAAGGACCCATCTCTTAAAC-3’	59	584
TGFBI_5R	5’-GTGATGGTGGAGATGACCTTAT-3’		
TGFBI_6F	5’-CTTTGGGACTATGCCTCTGTTG-3’	58	566
TGFBI_6R	5’-AAGCACACACAGCCTGATAG-3’		
TGFBI_7F	5’-AGCAGACAGACTCACCTTTG-3’	58	628
TGFBI_7R	5’-CAGGTGGTATGTTCATCTTGGA-3’		
TGFBI_8F	5’-AGGAAGTTGAGGTTCAAAGAGG-3’	60	609
TGFBI_8R	5’-ATTTGCAGTGGCCGAGAA-3’		
TGFBI_9F	5’-GGTTGTTGACTCACGAGATGA-3’	59	380
TGFBI_9R	5’-TTGGTTGAGCTGAGTGGAAG-3’		
TGFBI_10F	5’-CTTGGTTTCTCAATCCCTGTTTC-3’	60	659
TGFBI_10R	5’-GAAGATACAGCAGAAGAGAGAAGG-3’		
TGFBI_11F	5’-GTCAATGGACCTCATGTGTAACT-3’	59	531
TGFBI_11R	5’-GCACTGCTAGTGTTAATTGAATCC-3’		

Supplementary Table 1. Primers for *VSX1, TGFBI* and *ZEB1* amplification. (continued)

Primer[a]	Sequence	TM(°C)	Product size(bp)
TGFBI_12F	5'-CTGCCCTCCCAGAATCATATC-3'	60	598
TGFBI_12R	5'-CCGCCCACTCTTTCACTAAT-3'		
TGFBI_13F	5'-CCATTAGACAGATTGTGGGTCA-3'	60	544
TGFBI_13R	5'-CAGATGCTCTATTCCCTTGTAAATC-3'		
TGFBI_14F	5'-CCTGGGCGACAAGATTGAAA-3'	63	411
TGFBI_14R	5'-CTCCCAATTCACTCTGCAATCA-3'		
TGFBI_15F	5'-CATTGCTCTTTGCGGAGTTG-3'	61	639
TGFBI_15R	5'-GGGAGTTGCCTTGGTTCTTTA-3'		
TGFBI_16F	5'-ACTCCCTGGTGCCTATAAGT-3'	57	318
TGFBI_16R	5'-TGCTGTTCTGACTCCAAAGG-3'		
TGFBI_17F	5'-GAACATCGGGCCTTGAGTAA-3'	59	482
TGFBI_17R	5'-GTCCTCTCTGGAAGTGAATGTC-3'		
ZEB1_1F	5'-GTAAAGCCGGGAGTGTCGTA-3'	60	422
ZEB1_1R	5'-GCGGAGAGAGACCAGGTAAG-3'		
ZEB1_2F	5'-CATTGAATTACAATCTGTTTTAAGCAT-3'	58	406
ZEB1_2R	5'-TTGATTTCAAACTTTTCATCCA-3'		
ZEB1_3F	5'-CCTTTTCAGATTTCGGGAAG-3'	58	452
ZEB1_3R	5'-TGTAGTGCACCATATGCCTAAGT-3'		
ZEB1_4F	5'-TTCTGCAGATTCAAGAACAATCA-3'	60	452
ZEB1_4R	5'-TGCATGGTCATCATAGTGTTCC-3'		
ZEB1_5F	5'-GTGGGTAGCACAATATCTGG-3'	55	433
ZEB1_5R	5'-AGGCTGCAGATATAGCACTG-3'		
ZEB1_6F	5'-CAACCATCAGGCTCACAAAA-3'	59	433
ZEB1_6R	5'-TGATTAGGAAATCTGGAGTATGTCA-3'		
ZEB1_7_1F	5'-CCGCTTGTTTTAGGGAAATG-3'	60	747
ZEB1_7_1R	5'-AGTTGGCTAGGCTGCTCAAG-3'		
ZEB1_7_2F	5'-AGCCATCAGTCTTCCTTTGG-3'	59	814
ZEB1_7_2R	5'-CTCTTCTTGTGCACCCTCAG-3'		
ZEB1_7_3F	5'-GATCAACCACCAATGGTTCC-3'	60	717
ZEB1_7_3R	5'-CCCTGGATTAGCAAACAACC-3'		
ZEB1_8F	5'-TCAGTGTGCTTGCTTTGGTC-3'	60	450
ZEB1_8R	5'-GAGCCAGACCTTGTCTCAAAA-3'		
ZEB1_9F	5'-GAGTTTGGGACCTGGAAATG-3'	59	808
ZEB1_9R	5'-TGTTTCCATGAAAAGCAAGG-3'		

[a]F=Forward primer, R=Reverse primer.
Tm=Melting temperature.

Supplementary Table 2. Candidate pathogenic genes of keratoconus.

CAST	CDH11	COL1A1	COL27A1	COL4A1
COL4A3	COL4A4	COL5A1	COL6A1	COL8A1
COL8A2	CRB1	CRX	DOCK9	FNDC3B
FOXO1	HGF	IL1A	IL1B	IL1RN
LOX	MAN2C1	MIR184	MMP-9	MPDZ
MPDZ-NF1B	NTRK1	NUB1	PRDM5	RAB3GAP1
RXRA	SLC4A11	SOD1	SPARC	TGFBI
TUBA3D	VSX1	WNT10A	ZEB1	ZNF469

Supplementary Table 3. Bioinformatics data of variants from Sanger sequencing and WES in the four probands.

Family	method	Source	Gene	Amino Acid Change	1000G	ExAC	gnomAD	Mutation Taster[a]	Polyphen2[b]	SIFT[c]
F1	**WES/Sanger**	**Maternal**	**TGFBI**	**c.471C>G:p.D157E**	**0.0008**	**0.00009**	**0.00012**	**D**	**P**	**T**
	WES	Maternal	IL1RN	c.370G>A:p.A124T	0.0048	0.003	0.00304	N	B	T
	WES	Paternal	FOXO1	c.1532C>T:p.A511V	0.0036	0.0014	0.00141	D	B	T
F2	**WES/Sanger**	**Maternal**	**TGFBI**	**c.1870G>A:p.V624M**	**0.001**	**0.0003**	**0.00024**	**D**	**D**	**D**
	WES	Paternal	ZNF469	c.1471G>A:p.A491T	0.00998	0.0014	0.00844	N	B	T
	WES	Paternal	ZNF469	c.3430C>T:p.R1144C	NI	NI	0.00005	N	B	D
F3	**WES/Sanger**	**Paternal**	**VSX1**	**c.758-765delTCAACTCC p.L253Rfs*18**	**NI**	**0.00013**	**0.0002**	**D**	**NI**	**NI**
	WES	Paternal	COL4A3	c.3627G>A:p.M1209I	0.009	0.0045	0.0027	N	B	T
	WES	Maternal	ZNF469	c.3668C>T:p.A1223V	0.0014	0.0002	0.0003	N	B	T
G1	**WES/Sanger**	**Maternal**	**TGFBI**	**c.805C>T:p.L269F**	**0.0024**	**0.0018**	**0.00165**	**D**	**D**	**D**
	**WES/Sanger**	**Paternal**	**TGFBI**	**c.1998G>C:p.R666S**	**0.0008**	**0.0016**	**0.00117**	**A**	**B**	**T**
	WES	Maternal	IL1B	c.28G>A:p.E10K	0.0002	0.0001	0.00012	N	D	D
	WES	Maternal	SLC4A11	c.941C>T:p.A314V	0.0012	0.0015	0.00138	N	B	T

[a]MutationTaster: A: known to be deleterious; D: probably deleterious; N: probably harmless; P: known to be harmless.
[b]Polyphen2: D: probably damaging; P: possibly damaging; B: benign.
[c]SIFT: D: Deleterious; T: tolerate.
Variant in **bold font** was identified by Sanger sequencing in VSX1, TGFBI and ZEB1 genes.
WES=whole exome sequencing; Het=heterozygosis; NI=no information.

Supplementary Table 4. Data of BAD maps of asymptomatic parents.

Parameter	The asymptomatic parents without mutation						The asymptomatic parents with mutation							
	F1:I:1 OD	F1:I:1 OS	F2:I:1 OD[a]	F2:I:1 OS	F3:I:2 OD	F3:I:2 OS	F1:I:2 OD	F1:I:2 OS	F2:I:2 OD	F2:I:2 OS	F3:I:1 OD	F3:I:1 OS	G1:I:2 OD	G1:I:2 OS
TP (μm)	512	519	490	497	516	522	497	509	500	498	515	508	484	478
F.Ele.Thb (μm)	4	3	5	3	3	3	3	4	1	2	1	0	3	4
B.Ele.Thb (μm)	9	6	7	2	5	9	8	5	12	16	17	10	9	13
PPIavg	1.12	1.02	1.29	1.01	1.02	1.07	1.22	1.04	1.2	1.25	1.41	1.26	0.94	1.13
ARTmax	331	335	276	394	432	409	292	393	326	319	295	335	403	347
Df	-0.8	0.1	1.08	0.99	0.33	1.01	1.54	1.94	-0.94	0.24	-1.26	-1.39	0.81	1.49
Db	-0.23	-0.8	-0.48	-1.16	0.09	1.04	0.16	0.41	1.49	1.72	1.59	1.38	0.23	1.13
Dp	1.48	0.77	2.6	0.69	0.79	1.1	2.14	0.93	1.97	2.32	3.42	2.38	0.27	1.55
Dt	0.79	0.55	1.52	1.28	0.66	0.48	1.27	0.89	1.16	1.24	0.7	0.9	1.71	1.92
Da	1.43	1.4	1.93	0.86	0.51	0.72	1.79	0.87	1.48	1.54	1.76	1.4	0.77	1.29
D	1.66	1.61	2.3	1.35	1.14	1.61	2.35	1.87	2.07	2.6	2.43	2	1.67	2.39

[a]The right eye of F2: I:1 had pterygium, corneal macula, and explicit history of trauma, so the corneal data of right eye was excluded from our study.
[b]Diameter of reference zone of best fit sphere (BFS) = 8 mm.
OD=right eye; OS=left eye.
